# Ectopic localization of FOXO3a protein in Lewy bodies in Lewy body dementia and Parkinson's disease

**DOI:** 10.1186/1750-1326-4-32

**Published:** 2009-07-23

**Authors:** Bo Su, Haihua Liu, Xinglong Wang, Shu G Chen, Sandra L Siedlak, Eisaku Kondo, Raymond Choi, Atsushi Takeda, Rudy J Castellani, George Perry, Mark A Smith, Xiongwei Zhu, Hyoung-gon Lee

**Affiliations:** 1 Department of Pathology, Case Western Reserve University, Cleveland, Ohio 44106, USA; 2 Department of Pathology, Okayama University Graduate School of Medicine and Dentistry, Okayama 700-8558, Japan; 3 Department of Pathology, University of Maryland, Baltimore, Maryland 21201, USA; 4 Division of Neurology, Department of Neuroscience, Tohoku University Graduate School of Medicine, Sendai, Japan; 5 UTSA Neurosciences Institute and Department of Biology, College of Sciences, University of Texas at San Antonio, San Antonio, Texas 78249, USA

## Abstract

Lewy bodies and Lewy neurites constitute the cardinal neuropathological features of both Parkinson's disease (PD) and Lewy body dementia (LBD). Whereas α-synuclein has been found to be the major component of the Lewy body, the mechanisms by which neurons degenerate, as well as basic mechanisms involved in the formation of α-synuclein-related inclusions, remain obscure. We have suggested previously that potential mechanisms are likely to leave a "molecular signature" or protein adduct within the Lewy body, and have found examples of such signatures in previous studies. In this study, we demonstrate increased FOXO3 in association with Lewy bodies and Lewy neurites in LBD and PD brain tissue. Since FOXO proteins are involved in several pathways responsible for the regulation of cell death, cell proliferation, and cell metabolism, the ectopic localization of FOXO3 to Lewy bodies provides evidence that aberrations in basic cellular biochemistry may contribute to inclusion formation, which is likely more complex than a simple "gain of function" toxicity as is commonly opined. In light of the known interaction of FOXO3 and 14-3-3, basic protein-protein interaction between these proteins and α-synuclein may be key.

## Background

Parkinson's disease (PD) is the second most common age-related neurodegenerative disease after Alzheimer's disease (AD) [1,2]. Like many other neurodegenerative diseases, PD and Lewy body dementia (LBD) are increasingly recognized as disorders of protein aggregation and inclusion body formation [3]; in particular, PD is defined by the presence of Lewy bodies and Lewy neurites [4,5]. Cortical Lewy bodies also constitute the defining neuropathological characteristics of LBD, a common form of dementia that exists in a "pure" form or overlaps with AD neuropathology [4]. α-Synuclein is the major component of the Lewy body in PD and LBD [6] and is currently used as a diagnostic marker of Lewy bodies. Mutations in α-synuclein are also described in a subset of familial PD kindreds, whereas aggregated α-synuclein occurs in both familial and sporadic PD lesions [7]. Besides α-synuclein, many molecules such as 14-3-3 protein and ubiquitin have been found in Lewy bodies [8-10]. It is also suggested that 14-3-3 protein which shares amino acid sequence homology with α-synuclein may be associated with Lewy body formation [8].

It is generally accepted that progressive, irreversible and regionally specific neurodegeneration, with Lewy bodies and Lewy neurites are the essential pathological hallmarks of idiopathic PD. The precise etiology, however, is unclear. One of the leading hypotheses suggests that oxidative stress and generation of reactive oxygen species damages macromolecules, resulting ultimately in cell death [11]. In fact, Lewy bodies themselves have been shown to contain adducts induced by oxidative stress [12].

The forkhead box transcription factor, class O (FOXO) is the mammalian homologue of DAF-16, which is known to regulate life span of *Caenorhabditis elegans *[13,14]. In mammals, the FOXO class of transcription factors are key players in the regulation of cell-fate decisions, such as cell death, cell proliferation, and cell metabolism [15]. It has been reported that phosphorylation/dephosphorylation of FOXO protein, which results in the translocation between cytoplasm and nucleus, is a major regulatory mechanism of FOXO-dependent-transcription [16]. A recent study indicated that three FOXO family members including FOXO1, FOXO3, and FOXO4 play essential roles in the response to physiologic oxidative stress in hematopoietic stem cells of experimental mice [17]. In the present study, we examined the expression of FOXO3a (also known as FKHRL1) in brain tissue from cases of PD and LBD.

## Methods

### Brain tissue

Hippocampal tissue with adjacent temporal cortex from patients with "pure" LBD (n = 3, ages 68–78 years), LBD plus AD pathology (AD/LBD) (n = 4, ages 59–84 years), brainstem from PD cases (n = 4, ages 53–108 years), and age-matched controls (n = 3, ages 66–86 years) with similar postmortem intervals (LBD, AD/LBD and PD: 4–15 h; controls: 6.25–22 h), were fixed either in formalin or in methacarn (methanol:chloroform:acetic acid; 60:30:10) at 4°C overnight. Following fixation, tissue was dehydrated through ascending ethanol, embedded in paraffin, and 6-μm sections placed on silane-coated slides.

### Immunohistochemistry

Immunohistochemistry was performed by the peroxidase anti-peroxidase protocol essentially as described previously [18,19]. All slides were randomized and blinded with regards to age and disease status prior to immunohistochemical staining and subsequent analysis. Briefly, slides were immersed in xylene, hydrated through graded ethanol solutions, and endogenous peroxidase activity eliminated by incubation in 3% hydrogen peroxide for 30 min. To reduce non-specific binding, sections were incubated for 30 min in 10% normal goat serum in Tris-buffered saline (TBS; 50 mM Tris-HCl, 150 mM NaCl, pH 7.6). After rinsing briefly with 1% normal goat serum in TBS, the sections were incubated overnight at 4°C with either rabbit polyclonal antibody to FKHRL1 (FOXO3a) (1:100) [20], mouse monoclonal antibody to α-synuclein (1:100; Abcam, Cambridge, MA) or rabbit polyclonal antibody to 14-3-3 β (1:100; Santa Cruz Biotechnology, Santa Cruz, CA). Sections were sequentially thereafter incubated with either goat anti-rabbit or goat anti-mouse antisera (ICN, Costa Mesa, CA) followed by species-specific peroxidase anti-peroxidase antibody (ICN, Costa Mesa, CA). Antibodies were localized using 3-3'-diaminobenzidine as a chromogen (Dako Corp, Carpinteria, CA). In some cases, antigen retrieval using Biocare Medical decloaking chamber was applied before the incubation of the primary antibody.

## Results

**Figure 1:**
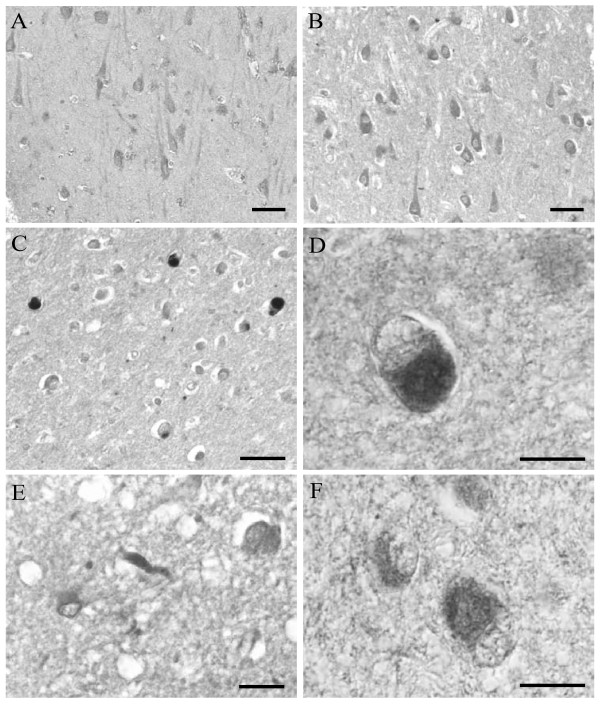
**In hippocampal neurons, FOXO3a is present at similar levels in control (A), and cases diagnosed with LBD (B)**. Cortical Lewy bodies, however, exhibit intense immunolabeling for FOXO3a, in cortical regions from all cases diagnosed with pure LBD (C), and with AD/LBD (D). Lewy neurites also accumulate FOXO3a (E). Significantly, cortical Lewy bodies also demonstrate increased 14-3-3 (F). Scale bars = 50 μm (A-C); 20 μm (D-F).

The cellular localization of FOXO3a was examined in the hippocampus of LBD, AD/LBD, and age-matched control patients by immunohistochemical techniques using an antibody to FOXO3a. Whereas immunoreactivity of FOXO3a localized to the cytoplasm similarly in hippocampal neurons in control cases (Fig. 1A) as well as in cases of LBD (Fig. 1B), FOXO3a strongly and specifically localized to cortical Lewy bodies in all cases of pure LBD (Fig. 1C) and AD/LBD (Fig. 1D, case of age 64). Lewy neurites also contained FOXO3a in LBD and AD/LBD (Fig. 1E). Immunostaining these same cases for 14-3-3 protein also revealed specific localization in cortical Lewy bodies (Fig. 1F, case of age 59).

**Figure 2:**
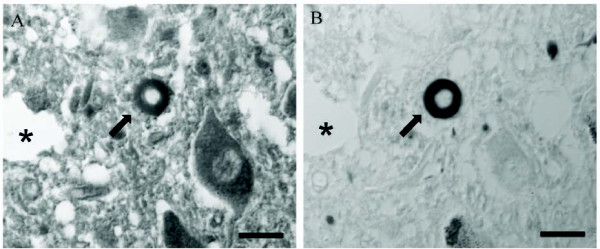
**Adjacent serial sections of brainstem of a case of PD immunostained for FOXO3a (A) and α-synuclein (B)**. Co-localization of FOXO3a and α-synuclein in classical Lewy bodies (arrows) was observed. *Landmark vessel. Scale bars = 20 μm.

The significant overlap between the immunoreactivity to α-synuclein and FOXO3a further confirms the specific localization of FOXO3a in cortical Lewy bodies as well as in classical Lewy bodies in the brainstem of cases with PD (Fig. 2). Most Lewy bodies were found to be positive for α-synuclein and FOXO3a on adjacent serial sections, indicating the presence of FOXO3a in Lewy bodies in PD brain.

## Discussion

The localization of FOXO3a to Lewy bodies and Lewy neurites in PD and LBD indicates that FOXO3a is a potential component of Lewy bodies. While Lewy bodies are known also to be comprised of α-synuclein and ubiquitin [21] and occur in a variety of clinical disease states [7,22-24], the mechanism of α-synuclein accumulation in the Lewy body and its role in disease is poorly understood [7]. The finding of FOXO3a in intimate association with PD/LBD lesions suggests that LB and Lewy neurite formation encompasses more basic cellular pathophysiology than simple "gain of function" toxicity. Given the role of FOXO in such processes as phosphorylation, acetylation/deacetylation, ubiquitination, and protein-protein interactions [25], the potential of multiple hits cannot be discounted [26,27]. In fact, FOXO3a has been shown to trigger the death of motoneurons in mice with the translocation from cytoplasm to nucleus [28], and a recent study also showed oxidative stress induces neuronal necrosis by activating FOXO3 [29].

Interestingly, however, several lines of evidence now indicate that α-synuclein accumulation is fundamentally protective [30,31]. In our own studies, α-synuclein aggregation occurs in concert with microtubule polymerization and that an aggregate, once formed, is cytoprotective response against noxious stimuli [32,33]. This is compatible with the once controversial concept that neurodegenerative disease lesions are markers of pathogenic disease response rather than indicators of etiology [34,35]

Oxidative stress is believed to be an important factor in cell death induction and considerable evidence has accumulated that oxidative stress is involved in the pathogenesis of PD and α-synuclein aggregation [36-38]. As a transcription factor, it is necessary for FOXO3a to locate in nucleus to play its cell death-inducing role. In the present study, the high level expression of FOXO3a protein in an inclusion body in the neuronal cytoplasm suggests that FOXO3a may not reach the nucleus (e.g., to affect its known function in the apoptotic pathway). This finding adds further evidence to the notion that Lewy bodies may protect affected neurons from death and furthers the notion that profound alterations in cytoplasmic-nuclear trafficking are a key element of the neurodegenerative process [39,40]. The finding of the specific isoform FOXO3a in neurodegenerative disease as presented in this work, as well as its role in attenuating amyloid-like pathology in mouse models [41], clearly supports the investigation of the other members of the FOXO family transcription factor in aging and disease.

14-3-3 is a family of dimeric proteins that can modulate interaction between proteins and they are involved in cell signaling, regulation of cell cycle progression, intracellular trafficking/targeting, cytoskeletal structure and transcription [42,43]. In previous studies, phosphorylated FOXO3a has been shown to bind to 14-3-3 protein, causing cytoplasmic retention of phosphorylated FOXO3 and inhibition of FOXO3-induced transcriptional activation [16]. Interestingly, 14-3-3 protein has also been localized to Lewy bodies [8]. Therefore, while the mechanism of accumulation of FOXO3a in Lewy bodies awaits further study, a direct interaction between FOXO3a and 14-3-3 protein remains a distinct possibility. Since α-synuclein shares physical and functional homology with 14-3-3 proteins [44], it is reasonable to speculate that FOXO3a, α-synuclein, and 14-3-3 protein may form a complex, preventing the dephosphorylation and translocation of FOXO3a, and promote cell survival. This potential protein interaction is the subject of ongoing studies in our laboratory.

## Conclusion

Our study demonstrates the localization of FOXO3a protein to Lewy bodies and Lewy neurites, suggesting a role of FOXO3a in the morphogenesis of inclusions in synucleinopathies. Given the pleiotrophic effects of FOXO3a in cellular pathophysiology, our results further the increasingly important concept that inclusion formation is a complex process, favoring an adaptive disease response over a primary deleterious process. Further studies investigating direct protein interaction *in vitro *and *in vivo *are in progress.

## Abbreviations

(AD): Alzheimer's disease; (FOXO): forkhead box transcription factor, class O; (LBD): Lewy body dementia; (PD): Parkinson's disease; (TBS): Tris-buffered saline.

## Competing interests

The authors declare that they have no competing interests.

## Authors' contributions

HL, SLS, RC, and BS carried out the experiments. XWZ, RJC, MAS and HGL designed the study, analyzed data, and wrote the manuscript. SGC, XW, EK, AT, and GP participated in the design of the study and helped analyze the data. All authors read and approved the final manuscript.
